# The Identification of Three Cancer Stem Cell Subpopulations within Moderately Differentiated Lip Squamous Cell Carcinoma

**DOI:** 10.3389/fsurg.2017.00012

**Published:** 2017-03-06

**Authors:** Rachna Ram, Helen D. Brasch, Jonathan C. Dunne, Paul F. Davis, Swee T. Tan, Tinte Itinteang

**Affiliations:** ^1^Gillies McIndoe Research Institute, Wellington, New Zealand; ^2^Wellington Regional Plastic, Maxillofacial and Burns Unit, Hutt Hospital, Wellington, New Zealand

**Keywords:** lip, oral cavity, squamous cell, carcinoma, cancer stem cell, cancer

## Abstract

**Aim:**

To identify and characterize cancer stem cells (CSCs) in moderately differentiated lip squamous cell carcinoma (MDLSCC).

**Method:**

MDLSCC samples underwent 3,3-diaminobenzidine (DAB) immunohistochemical (IHC) staining for squamous cell carcinoma marker EMA, CSC marker CD44 and embryonic stem cell markers NANOG, octamer-binding transcription factor 4 (OCT4), spalt-like transcription factor 4 (SALL4), sex-determining region Y-box 2 (SOX2), and phosphorylated signal transducer and activator of transcription 3 (pSTAT3). Immunofluorescent IHC staining was performed on two MDLSCC samples. Western blotting (WB) was used to confirm the expression of the aforementioned proteins and their transcription activation was investigated using NanoString and RT-qPCR.

**Results:**

IHC staining demonstrated the presence of (1) an EMA^+^/CD44^+^/SALL4^+^/NANOG^+^/pSTAT3^+^/SOX2^+^/OCT4^−^ CSC subpopulation within the tumor nests (TNs); (2) a CD44^+^/SALL4^+^/NANOG^+^/pSTAT3^+^/SOX2^+^/OCT4^−^ CSC subpopulation; and (3) a CD44^+^/SALL4^+^/NANOG^+^/pSTAT3^+^/SOX2^+^/OCT4^+^ CSC subpopulation within the stroma, between the TNs. NanoString and RT-qPCR confirmed the presence of mRNA for CD44, SALL4, STAT3, SOX2, and OCT4, and WB confirmed the presence of NANOG, pSTAT3, SOX2, and OCT4.

**Conclusion:**

This study demonstrates three putative CSC subpopulations within MDLSCC.

## Introduction

Lip cancer constitutes a subsite of oral cavity cancer with more than 80% being squamous cell carcinoma (SCC) (1). There is a higher incidence of lip SCC in males in North America (12.7/100,000 per annum), Europe (12.0/100,000 per annum), and Oceania (13.5/100,000 per annum) (2) and the incidence is rising among females (2).

The etiological factors for oral cavity SCC (OCSCC) including lip SCC include allelic imbalance involving tumor suppressor genes (3), oncogenes (3), and carcinogen metabolizing enzymes (4), and the risk factors include tobacco use (5), immune deficiency (6), UV exposure (6), and human papilloma virus infection (7, 8). An overall 5-year survival rate of 88–97% has been reported (1, 9), and this is reduced to 78% in the presence of nodal metastasis and local recurrence (9–11). While surgery and radiotherapy are equally effective for the treatment of early lip SCC, combined treatment is required for advanced lesions (12).

There is growing evidence supporting the hierarchical model of carcinogenesis, which proposes that the development, growth, and spread of cancer are driven by a small population of cancer stem cells (CSCs) (13, 14). Various methods used for identification of CSCs in OCSCC include Hoechst dye exclusion, sphere forming assays, aldehyde dehydrogenase activity, and CSC marker identification (such as CD44, CD133, E-cadherin, keratins, and integrins) (14–16). More recent studies have utilized the embryonic stem cell (ESC) markers, sex-determining region Y (SRY)-box 2 (SOX2) (17), octamer-binding transcription factor 4 (OCT4) (17, 18), phosphorylated signal transducer and activator of transcription 3 (pSTAT3) (19), spalt-like transcription factor 4 (SALL4) (20), and Homeobox protein NANOG (18) to identify CSCs within OCSCC (21, 22).

CD44 is a transmembrane glycoprotein, a receptor for the glycosaminoglycan hyaluronan (23), and a CSC marker associated with cell proliferation, migration, and differentiation (24). SOX2 is a member of the SOX (SRY-related HMG Box) gene (25), encoding transcription factors with a single HMG DNA-binding domain and its suppression is considered vital to maintain stem cell pluripotency (17, 26). OCT4 is a POU domain transcription factor that works synergistically with SOX2 to regulate ESC pluripotency (27). SALL4 is an ESC marker that plays a role in multiple cancer types by regulating proliferation, apoptosis, chemoresistance, and maintenance of CSCs (23, 28), as well as modulating expression of pSTAT3, which is required for tumor formation, growth, and suppression of apoptosis (29). NANOG is a transcription factor that plays an essential role in maintaining stemness of ESC and controls cell proliferation, migration, and invasion (27, 30).

We have recently identified and characterized CSC subpopulations within oral tongue (21) and buccal mucosal (22) SCC. Although there are a number of reports on the presence and role of CSCs in OCSCC (21), there is paucity of data on the presence of CSC in lip SCC. This study aimed to identify and characterize the CSC subpopulations within moderately differentiated lip SCC (MDLSCC) using the SCC marker EMA, the CSC marker CD44, and the ESC markers SOX2, OCT4, pSTAT3, SALL4, and NANOG, at both the transcriptional and translational levels.

## Materials and Methods

### Tissue Samples

Previously untreated primary MDLSCC samples from one female and nine male patients, aged 46–94 years (mean, 64.4 years), were sourced from the Gillies McIndoe Research Institute and used for this study, which was approved by the Central Health and Disabilities Ethics Committee (ref. no. 12/CEN/74).

### Histochemical and Immunohistochemical (IHC) Staining

Hematoxylin and eosin (H&E) staining was performed on 4-μm thick formalin-fixed paraffin-embedded blocks of 10 MDLSCC samples that were subsequently analyzed by an anatomical pathologist (Helen D. Brasch) to confirm the presence of SCC and the histological grading. 3,3-Diaminobenzidine (DAB) IHC staining for NANOG (1:100; cat# ab80892, Abcam, Cambridge, MA, USA), SOX2 (1:200; cat# PA1-094, Thermo Fisher Scientific, Waltham, MA, USA), SALL4 (1:30; cat# CM385M-16, Cell Marque, Rocklin, CA, USA), pSTAT3 (1:100; cat# 9145, Cell Signaling Technology, Danvers, MA, USA), OCT4 (1:1,000; cat# ab109183, Abcam), CD44 (1:1,500; cat# MRQ-13, Cell Marque), and epithelial membrane antigen (EMA, ready-to-use; cat# PA0035, Leica) diluted with Bond™ primary antibody diluent (Leica AR9352) was performed on the tissue sections using the Leica Bond Rx auto-stainer (Leica) as previously described (21, 31).

Immunofluorescent (IF) IHC staining was performed on two representative MDLSCC samples from the original cohort used for DAB IHC staining to investigate protein co-expression. Vectafluor Excel anti-rabbit 594 (ready-to-use; cat# VEDK-1594, Vector Laboratories, Burlingame, CA, USA) and Alexa Fluor anti-mouse 488 (1:500; cat# A21202, Thermo Fisher Scientific) were used to detect combinations that included NANOG, SOX2, or pSTAT3 and VectaFluor Excel anti-mouse (ready-to-use; cat# VEDK2488, Vector Laboratories) and Alexa Fluor anti-rabbit 594 (1:500; cat# A21207, Thermo Fisher Scientific) were used to detect combinations that included OCT4 or SALL4. All IF IHC-stained slides were mounted in Vectashield HardSet Antifade mounting medium with 4′,6-diamidino-2-phenylindole (cat#H-1500, Vector Laboratories).

Positive human control tissues used for the primary antibodies were skin for EMA, tonsil for CD44, pSTAT3 and SOX2, and seminoma for NANOG, SALL4, and OCT4. Negative antibody control was performed on one MDLSCC sample per antibody staining run.

### Image Analysis

DAB IHC-stained slides were viewed using an Olympus BX53 light microscope (Tokyo, Japan) and the images were captured with the CellSens 2.0 software (Olympus). IF IHC-stained slides were viewed and the images were captured using an Olympus FV1200 biological confocal laser-scanning microscope and processed with the CellSens Dimension 1.11 software using 2D deconvolution algorithm (Olympus).

### NanoString Gene Expression Analysis

Total RNA was isolated and quantified as previously described (31) from six snap-frozen MDLSCC samples from the original cohort of 10 patients used for DAB IHC staining. They were subjected to the NanoString nCounter gene expression assay (NanoString Technologies, Seattle, WA, USA) by New Zealand Genomics (Dunedin, New Zealand). Briefly, total RNA was extracted using the MagJET RNA kit (Thermo Fisher Scientific) with the protocol adapted for tissue and run on a KingFisher Duo machine (Thermo Fisher Scientific). RNA samples were then quantitated on a Qubit^®^ 2.0 fluorometer (Thermo Fisher Scientific) and were subject to RNA integrity analysis *via* the 2100 Bioanalyzer Instrument (Agilent Technologies). Probes for the genes encoding CD44 (NM_001001392.1), NANOG (NM_024865.2), OCT4 (NM_002701.4), STAT3 (NM_139276.2), and the housekeeping genes glucuronidase beta (GUSB) (NM_000181.1), clathrin heavy chain (CLTC) (NM_4859.2), and hypoxanthine phosphoribosyltransferase 1 (NM_000194.1) were designed and manufactured by NanoString Technologies. Raw data were analyzed using nSolver™ software (NanoString Technologies) using standard settings and normalized against the housekeeping gene.

### RT-qPCR

Total RNA was isolated from six snap-frozen MDLSCC samples from the original cohort of 10 patients used for DAB IHC staining using the RNeasy mini Kit (Qiagen) with a DNase digest and the QIAcube system (Qiagen). Total RNA quantity and quality were assessed using NanoDrop 2000 (Thermo Fisher Scientific). Reverse transcription reactions were performed using the iScript Reverse Transcription Supermix for RT-qPCR (Bio-Rad, Hercules, CA, USA). The expression of stem cell markers was detected using gene-specific TaqMan primers-probe sets (SOX2: Hs01053049_s1; SALL4: Hs00360675_m1) with the Rotor-Gene Multiplex RT-PCR Kit (Qiagen). All measurements were performed in triplicate and relative mRNA expression was determined by the ΔΔCt method. GAPDH was used as the endogenous control and probe-specific gBlocks gene sequences (IDT technologies) were used as calibrators. All samples with a threshold cycle ≥35.0 were considered negative. Graphs were generated with Microsoft Excel and results are shown as relative expression.

### Western Blotting (WB)

Six snap-frozen MDLSCC samples from the original cohort of 10 patients included in DAB IHC staining underwent WB as previously described (32), using primary antibodies for NANOG (1:1,000; cat# ab47102, Abcam), OCT4 (1:2,000; cat# ab109183, Abcam), pSTAT3 (1:1,000; cat# 9145, Cell Signaling Technology), SOX2 (1:1,000; cat# PA1-094, Thermo Fisher Scientific), and β-actin (1:1,000; ab8226, Abcam). Two primary antibodies were used for SALL4 (1:1,000, cat# ab57577 and 1:1,000, cat# ab157172, both from Abcam). Secondary antibodies used were goat anti-rabbit horseradish peroxidase (HRP) conjugate (1:10,000; cat# A16110, Thermo Fisher Scientific) or Alexa Fluor^®^ 647 rabbit anti-mouse (1:2,000; cat# A21239, Thermo Fisher Scientific) as appropriate. HRP conjugated secondary antibody detection was achieved using Clarity™ Western ECL substrate (Bio-Rad). Membranes were imaged using a ChemiDoc MP imaging system (Bio-Rad).

## Results

### Histochemical and IHC Staining

Hematoxylin and eosin staining confirmed the diagnosis and histological grading of MDLSCC in all 10 samples. Staining patterns for the CSC markers in all 10 samples are shown in Table S1 in Supplementary Material.

**Figure 1:**
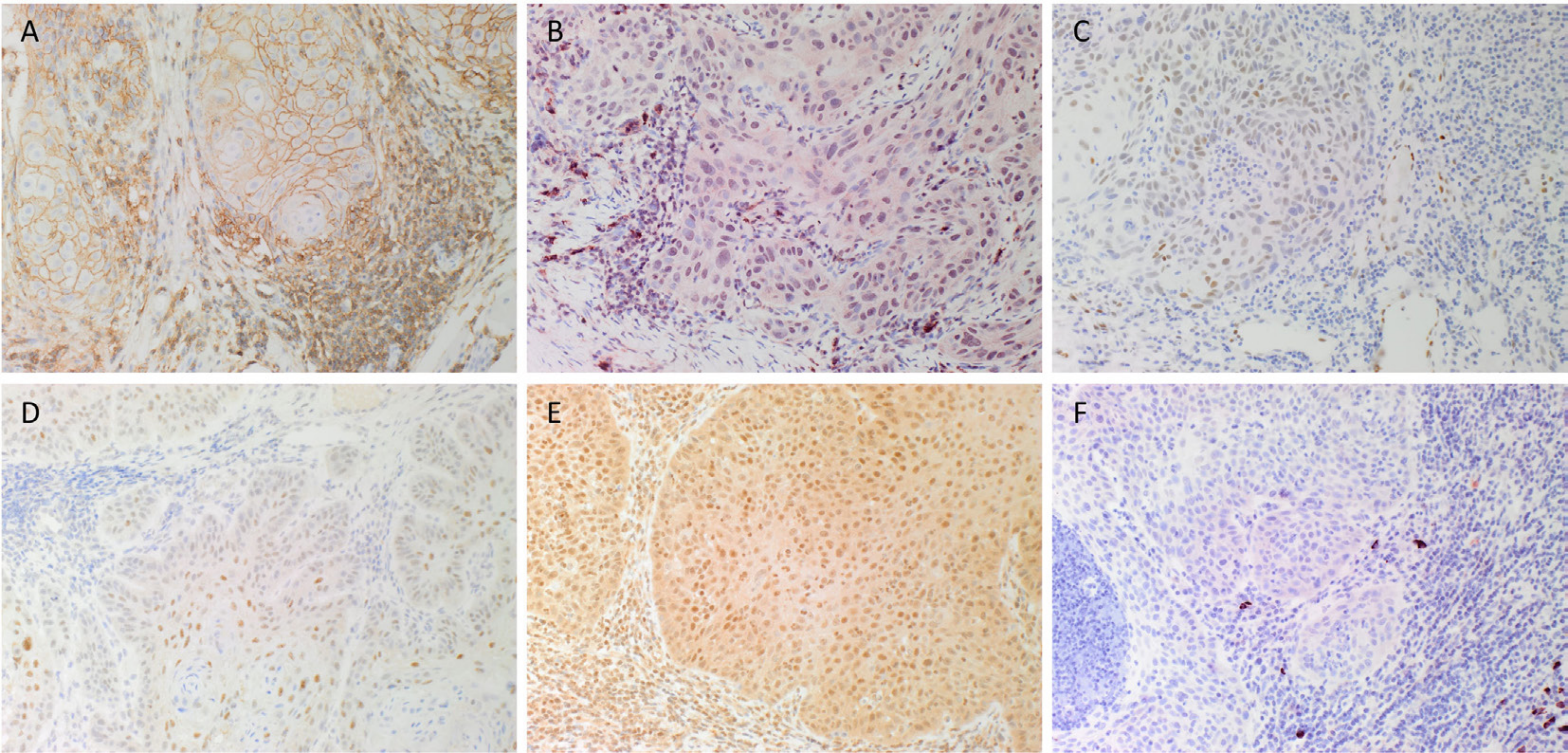
**Representative DAB immunohistochemical-stained sections of moderately differentiated lip squamous cell carcinoma demonstrating nuclear membrane staining of CD44 [(A), brown] on cells within the stroma and cell membrane staining on cells within the tumor nests (TNs)**. Nuclear staining of NANOG [**(B)**, red] was seen in cells within the TNs and the stroma. Patchy areas of weak staining for phosphorylated signal transducer and activator of transcription 3 [**(C)**, brown] was detected on cells within the TNs. Focal moderate expression of spalt-like transcription factor 4 [**(D)**, brown] on cells within the TNs and weak staining on cells within the stroma. Widespread and strong staining of sex-determining region Y-box 2 [**(E)**, brown] was seen on cells within the TNs and the stroma. Staining for octamer-binding transcription factor 4 [**(F)**, red] was limited to cells within the stroma. Original magnification: 200×.

DAB IHC staining demonstrated that some cells within the tumor nests (TNs) stained positively with the SCC marker EMA (data not shown), as expected. There was also membranous staining of the CSC marker CD44 (Figure 1A, brown) that was localized to cells within the TNs and the stroma. Staining of NANOG (Figure 1B, red), pSTAT3 (Figure 1C, brown), SALL4 (Figure 1D, brown), and SOX2 (Figure 1E, brown) was localized to cells within the TNs and the stroma. Immunoreactivity for OCT4 (Figure 1F, red) was primarily cytoplasmic and focal in cells within the stroma.

Expected staining patterns for CD44, NANOG, pSTAT3, SALL4, SOX2, and OCT4 (Figure S1 in Supplementary Material) were demonstrated in the respective positive controls. An appropriate negative control by the omission of the primary antibody in MDLSCC samples provided a control for the secondary antibody (Figure S2 in Supplementary Material).

**Figure 2:**
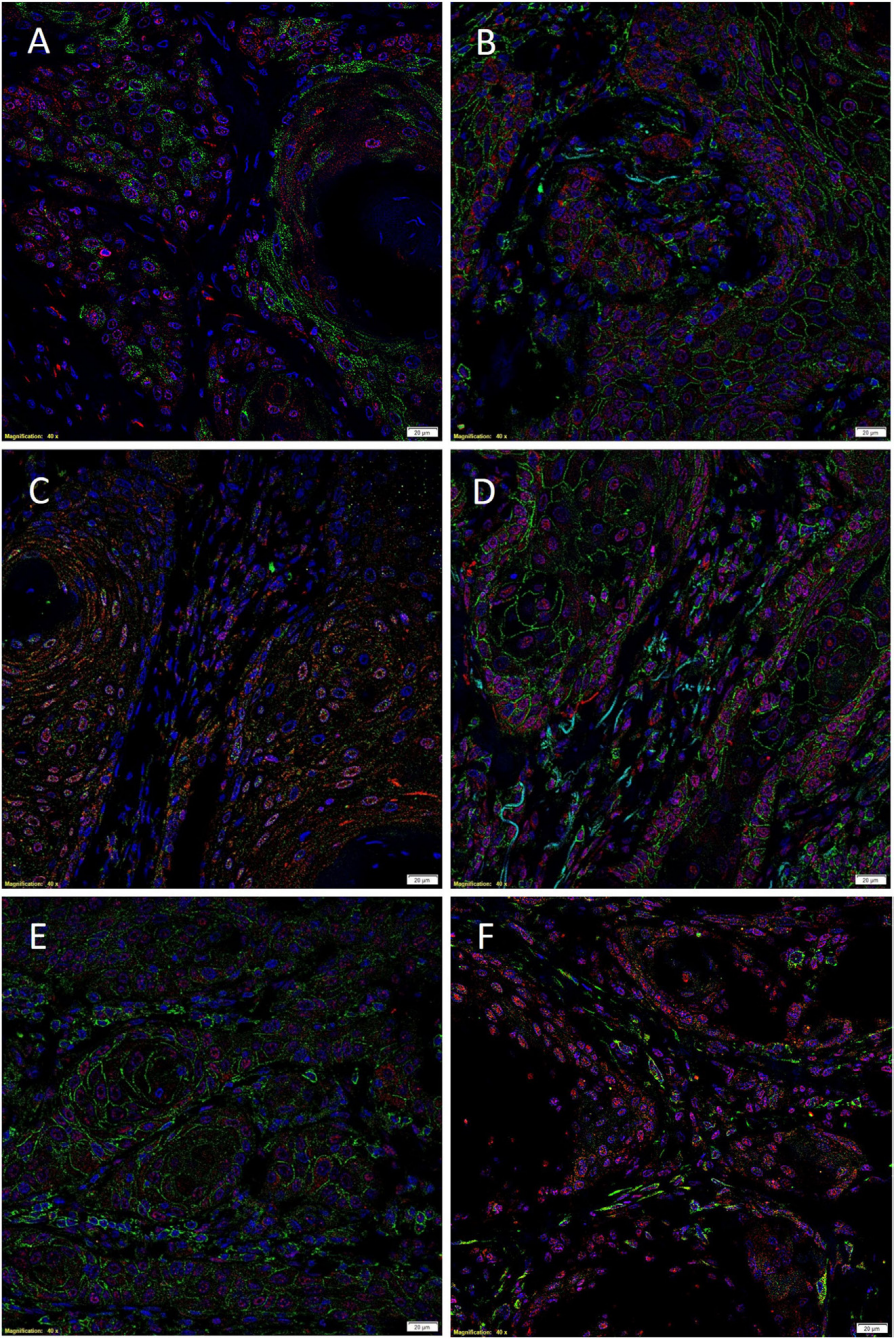
**Representative immunofluorescent immunohistochemical-stained sections of moderately differentiated lip squamous cell carcinoma demonstrating the expression of sex-determining region Y-box 2 (SOX2) [(A), red] by the EMA^+^ [(A), green] cells within the TNs and the stroma**. SOX2 [**(B,C)**, red] was expressed by cells within both the TNs and the stroma that expressed CD44 [**(B)**, green] and spalt-like transcription factor 4 [**(C)**, green]. Expression of NANOG [**(D)**, red] and phosphorylated signal transducer and activator of transcription 3 [**(E)**, red] was seen in cells within the TNs and the stroma that expressed CD44 [**(D,E)**, green]. Octamer-binding transcription factor 4 [**(F)**, green] was expressed in a proportion of cells within the stroma that expressed SOX2 [**(F)**, red]. Cell nuclei were counterstained with 4′,6-diamidino-2-phenylindole [**(A–F)**, blue]. Scale bars: 20 μm.

Immunofluorescent IHC staining performed on two representative MDLSCC samples from the original cohort of 10 patients used for DAB IHC staining demonstrated expression of the SCC marker EMA (Figure 2A, green) by cells within the TNs, while SOX2 (Figure 2A, red) was localized to cells within both the TNs and the stroma. Dual staining of SOX2 (Figures 2B,C, red) with CD44 (Figure 2B, green) and SALL4 (Figure 2C, green) showed that SOX2 was localized to cells within the TNs and the stroma that also expressed CD44 and SALL4. Co-staining of NANOG (Figure 2D, red) and pSTAT3 (Figure 2E, red) with CD44 (Figures 2D,E, green) demonstrated that the CD44^+^ cells within the TNs and the stroma also expressed NANOG and pSTAT3. Interestingly, the expression of OCT4 (Figure 2F, green) was confined only to a proportion of SOX2^+^ (Figure 2F, red) cells within the stroma. Images of the individual stains are presented in Figure S3 in Supplementary Material.

### NanoString Gene Expression Analysis

**Figure 3:**
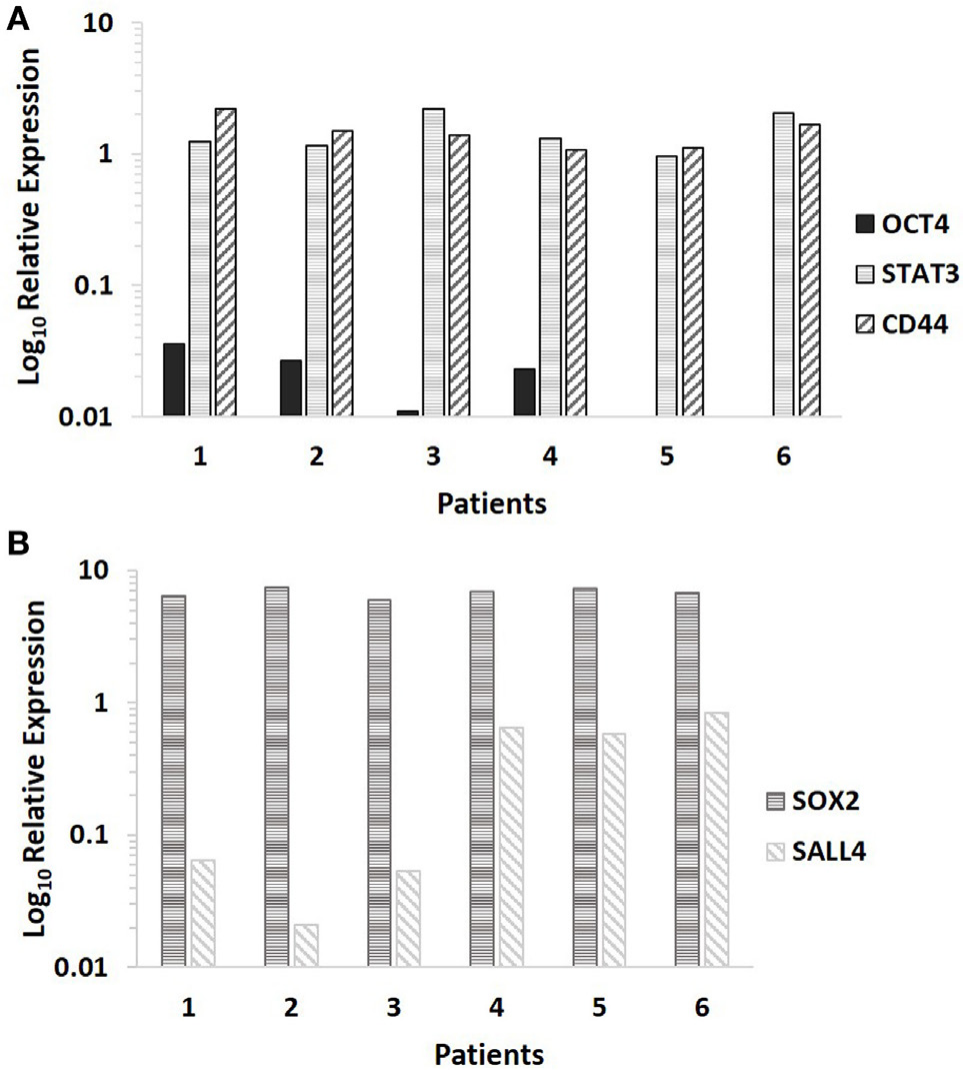
**Log_10_ relative expression of cancer stem cell-related mRNA transcripts in six moderately differentiated lip squamous cell carcinoma samples analyzed by NanoString (A) and RT-qPCR (B)**. Expression is depicted relative to the clathrin heavy chain housekeeping gene **(A)** and the GAPDH housekeeping gene **(B)**. CD44 and STAT3 were detected in all six samples, NANOG was undetectable in all six samples, while octamer-binding transcription factor 4 was detected in four out of six samples **(A)**. Sex-determining region Y-box 2 and spalt-like transcription factor 4 were detected in all six samples **(B)**.

NanoString transcriptional profiling of the six MDLSCC samples from the original cohort of 10 patients used for DAB IHC staining normalized against the housekeeping genes GUSB, CLTC, and HPRT1 confirmed the relative abundance of mRNA for CD44 and STAT3 in all the six samples. OCT4 was detected in four out of the six samples, while NANOG was undetectable in all six samples (Figure 3A).

### RT-qPCR

RT-qPCR analysis of six snap-frozen MDLSCC samples from the original cohort of 10 patients used for DAB IHC staining demonstrated abundance of mRNA transcripts for SOX2 and SALL4 in all six samples (Figure 3B).

### Western Blotting

**Figure 4:**
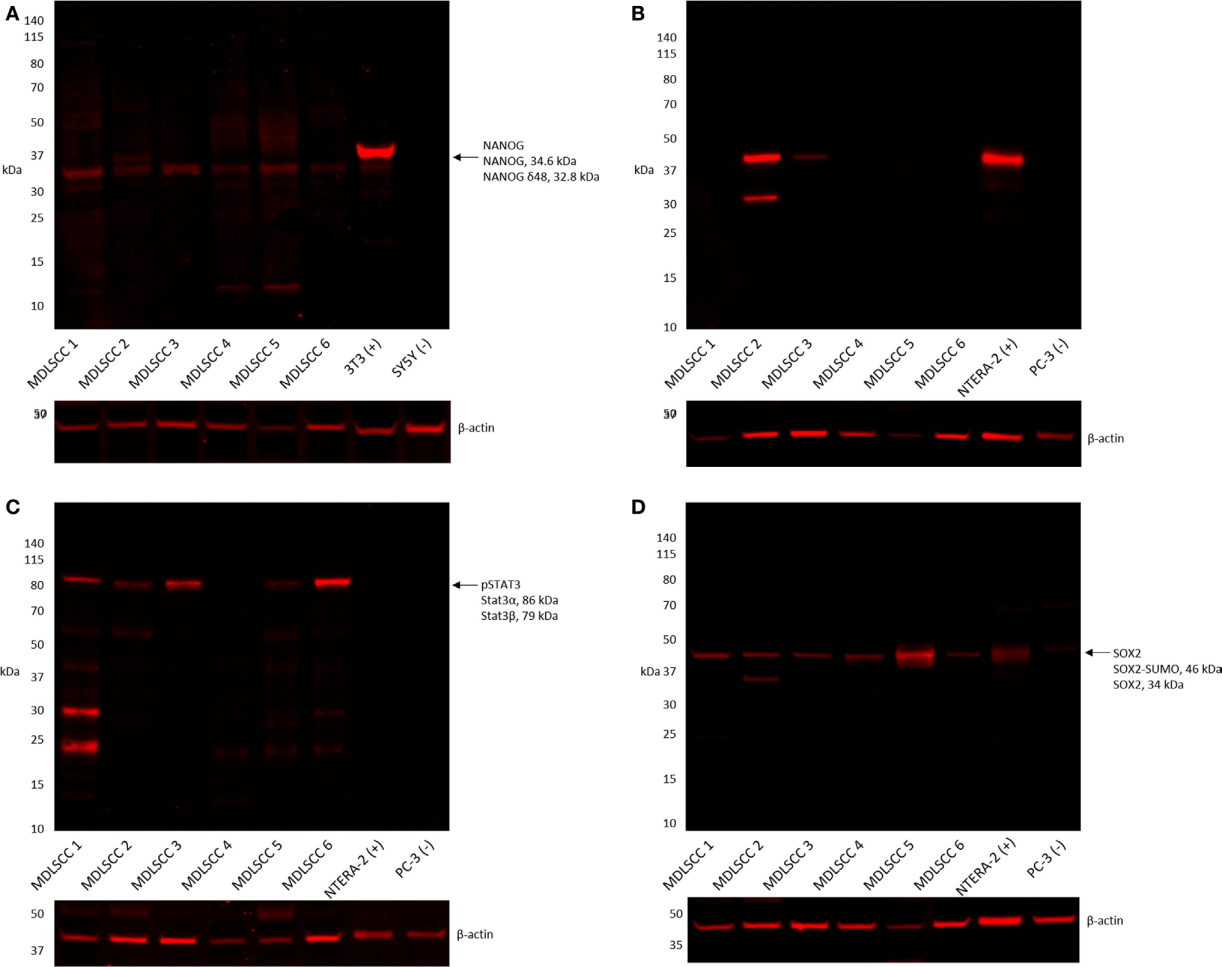
**Representative western blot analysis performed on six moderately differentiated lip squamous cell carcinoma samples demonstrating detection of NANOG across all six samples (A), octamer-binding transcription factor 4 in two samples (B), phosphorylated signal transducer and activator of transcription 3 in five samples (C), and sex-determining region Y-box 2 in all six samples (D)**.

Western blot analysis of six snap-frozen MDLSCC samples from the original cohort of 10 patients included in DAB IHC staining showed that NANOG was detected in all six samples (Figure 4A). OCT4 was detected in two of the six samples (Figure 4B), pSTAT3 was detected in five of the six samples (Figure 4C), and SOX2 was detected in all six samples (Figure 4D). A band corresponding to the expected size of SALL4 was undetectable by WB in any of the six samples (data not shown).

## Discussion

This study adds to the growing body of evidence for the presence of CSCs in OCSCC. Our study demonstrates the presence of three distinct CSC subpopulations within MDLSCC (1): a CD44^+^/SALL4^+^/NANOG^+^/pSTAT3^+^/SOX2^+^/OCT4^−^ subpopulation within the TNs (2); a CD44^+^/SALL4^+^/NANOG^+^/pSTAT3^+^/SOX2^+^/OCT4^−^, and (3) a CD44^+^/SALL4^+^/NANOG^+^/pSTAT3^+^/SOX2^+^/OCT4^+^ CSC subpopulation within the stroma of MDLSCC. It is exciting to speculate that the interactions between the CSCs within the TNs and the adjacent stromal environment may promote migration of CSCs away from the TNs, into the stroma, giving rise to the CSC subpopulations within the stroma, potentially *via* an epithelial–mesenchymal transition process (33–35). Alternatively, the primitive subpopulations within the stroma may represent normal “resident” stem cells, although this is a topic of further investigation.

The novel identification of more than one subpopulation of CSCs within MDLSCC parallels our recent findings in oral tongue (21) and buccal mucosa (22) SCC. Determination of which CSC subpopulation within the stroma possess the capacity for epithelial–mesenchymal transition (36) is a subject of further work, and we believe that a new paradigm opens up in the investigation of the biology of this tumor. The putative interplay between the stromal and TN CSC subpopulations raises the possibility of the CSC subpopulation within the TN giving rise to the CSC subpopulations within the stroma, which possess the ability to migrate away to establish local and distant TNs, although this is the subject of further investigation.

NANOG and SOX2 were expressed on cells within the TNs and the stroma. Overexpression of SOX2 in tumors has been correlated with increased tumor thickness and invasion, metastasis in esophageal cancer, drug resistance, and decreased survival in tumors such as breast cancer and lung adenocarcinoma (26, 37). Similarly, overexpression of NANOG is associated with unfavorable tumor features and poor survival of OCSCC patients (38).

Interestingly, OCT4 is expressed exclusively by the CSC subpopulation within the stroma. It is intriguing that the expression of OCT4 demonstrated by IHC staining in the stroma of all 10 MDLSCC samples was supported by NanoString and WB analyses in only two out of six samples. This may be due to sampling bias and/or antibody specificity. Investigating the expression of OCT4 in head and neck SCC samples of 119 patients, Koo et al. (39) demonstrate no staining and weak staining of OCT4 in 5% and 34% of the samples, respectively. Higher expression of OCT4 correlates significantly with poor histologic grade and worse overall and disease-specific survival in head and neck SCC (39).

Wu et al. (40) examined 156 pancreatic cancer tissue samples and found that high nuclear expression of pSTAT3 was associated with higher tumor grade and shorter median survival, compared to those with low expression of pSTAT3. Our IHC staining results showing weak expression of pSTAT3 by cells in both the TNs and the stroma is interesting and may reflect a tumor with relatively better prognosis. The relatively moderate expression of SALL4 as detected by IHC staining may be explained by the fact that only MDLSCC was analyzed in this study. High SALL4 expression levels have been correlated with poor overall survival of patients with aggressive tumors such as hepatocellular, endometrial cancer, gastric, and esophageal cancer (28).

This report demonstrates three putative subpopulations of CSCs within MDLSCC. It adds to the increasing support of the hierarchical concept of cancer. Further study may provide insights into the role CSCs may play in the biology of MDLSCC. It is exciting to speculate that CSCs may be a potential novel therapeutic target.

### Limitations

1. This study included a relatively small sample size. A larger study is needed to confirm the observed expression pattern.
2. Further work is needed on well and poorly differentiated lip SCC lesions to compare the expression patterns observed in this study.
3. Functional cell culture work is needed to demonstrate the ability for the three putative CSC subpopulations to form an orthoptic model for this cancer.

## Author Contributions

TI and ST formulated the study hypothesis and designed the study. RR, HB, TI, and ST interpreted the DAB and IF IHC data. JD performed WB analysis. JD, TI, PD, and ST interpreted the WB data. TI and ST interpreted the NanoString data. RR, TI, and ST drafted the manuscript. All authors commented on and approved the manuscript.

## Conflict of Interest Statement

The authors declare that the research was conducted in the absence of any commercial or financial relationships that could be construed as a potential conflict of interest. TI, PD, and ST are inventors of the PCT patent application (No. PCT/NZ2015/050108) cancer diagnosis and therapy.

## Abbreviations

DAB, 3,3-diaminobenzidine; CSC, cancer stem cell; ESC, embryonic stem cell; H&E, hematoxylin and eosin; IF, immunofluorescent; IHC, immunohistochemical; MDLSCC, moderately differentiated lip squamous cell carcinoma; OCSCC, oral cavity squamous cell carcinoma; SCC, squamous cell carcinoma; TN, tumor nest; WB, Western blotting.
