# Supplementary material for: The Identification of Three Cancer Stem Cell Subpopulations within Moderately Differentiated Lip Squamous Cell Carcinoma
Source: Front Surg. 2017 Mar 6;4:12. doi: 10.3389/fsurg.2017.00012 (PMC5337496; doi:10.3389/fsurg.2017.00012)
Supplement: Supplementary file 1 [file Data_Sheet_1.PDF]

Identification and Characterization of Cancer Stem Cells in Moderately Differentiated Lip Squamous Cell Carcinoma

[1]Rachna Ram MBBS, MSc, MMed, [1]Helen D Brasch MBChB, FRACPA, [1]Jonathan C Dunne PhD, [1]Paul F Davis PhD, [1,2]Swee T Tan◊* MBBS PhD FRACS, [1]Tinte Itinteang◊ MBBS, PhD

◊Equal senior authors

From the [1]Gillies McIndoe Research Institute, Wellington, New Zealand; and the [2]Wellington Regional Plastic, Maxillofacial & Burns Unit, Hutt Hospital, Wellington, New Zealand.

Supplementary Table 1 Individual DAB IHC Staining Patterns of ESC Markers in Moderately Differentiated SCC in 10 Patients

Patient	Markers					
	NANOG	CD44	OCT4	SOX2	pSTAT3	SALL4
1	T +<br>Stroma -	T ++<br>Stroma focal ++	T -<br>Stroma -	T +	T focal +	T focal +
2	T +<br>Stroma focal +	T ++<br>Stroma Diffuse ++	T -<br>Stroma focal ++	T +	T focal +	T focal +
3	T +<br>Stroma diffuse +	T ++<br>Stroma focal ++	T -<br>Stroma focal ++	T++	T focal +	T focal +
4	T +<br>Stroma focal ++	T ++<br>Stroma focal ++	T -<br>Stroma focal +	T++	T focal ++	T focal +
5	T -<br>Stroma focal +	T ++<br>Stroma diffuse ++	T-<br>Stroma focal ++	T+	T focal ++	T focal +
6	T+<br>Stroma focal ++	T ++<br>Stroma diffuse ++	T -<br>Stroma focal ++	T +	T focal ++	T focal ++
7	T ++<br>Stroma focal ++	T +++<br>Stroma diffuse ++	T -<br>Stroma focal ++	T++	T focal ++	T focal ++
8	T ++<br>Stroma focal ++	T ++<br>Stroma diffuse ++	T -<br>Stroma Focal ++	T+	T focal ++	T focal +
9	T +<br>Stroma diffuse ++	T ++<br>Stroma diffuse ++	T -<br>Stroma focal ++	T ++	T focal ++	T -
10	T -<br>Stroma focal ++	T ++<br>Stroma diffuse ++	T -<br>Stroma focal ++	T ++	T focal ++	T focal ++

T, tumor nests; +, positive; -, negativ

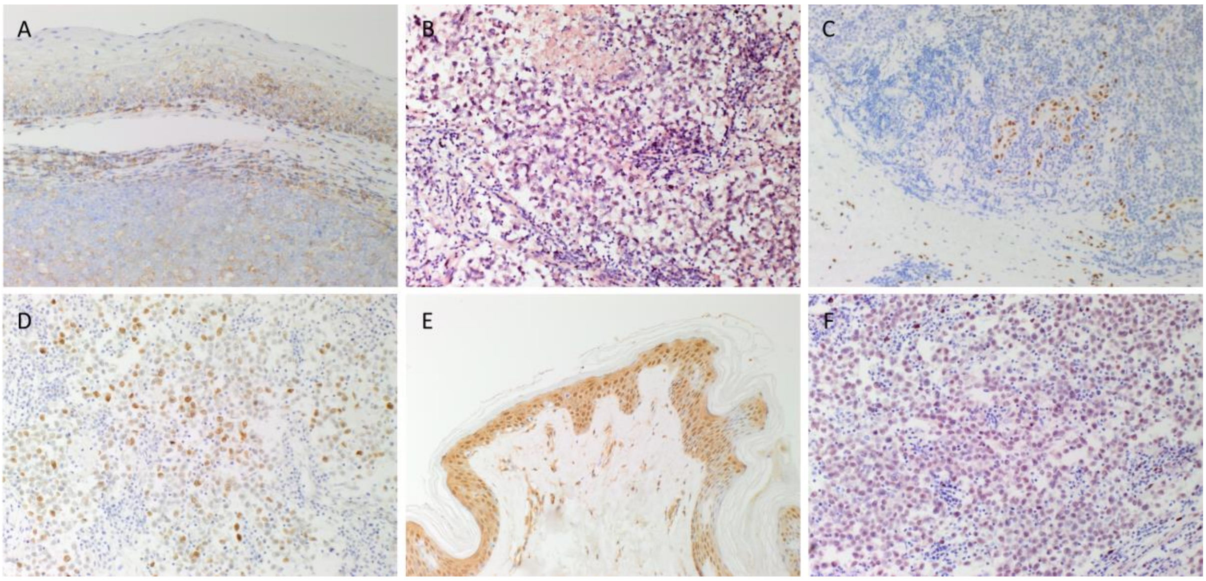

Figure S1. DAB IHC-stained sections of human tonsil for CD44 (**A**, brown), pSTAT3 (**C**, brown) and SOX2 (**E**, brown), and seminoma for NANOG (**B**, red), SALL4 (**D**, brown) and OCT4 (**F**, red). Cellular nuclei were counterstained with hematoxylin (**A-F**, blue). Original magnification: 200x.

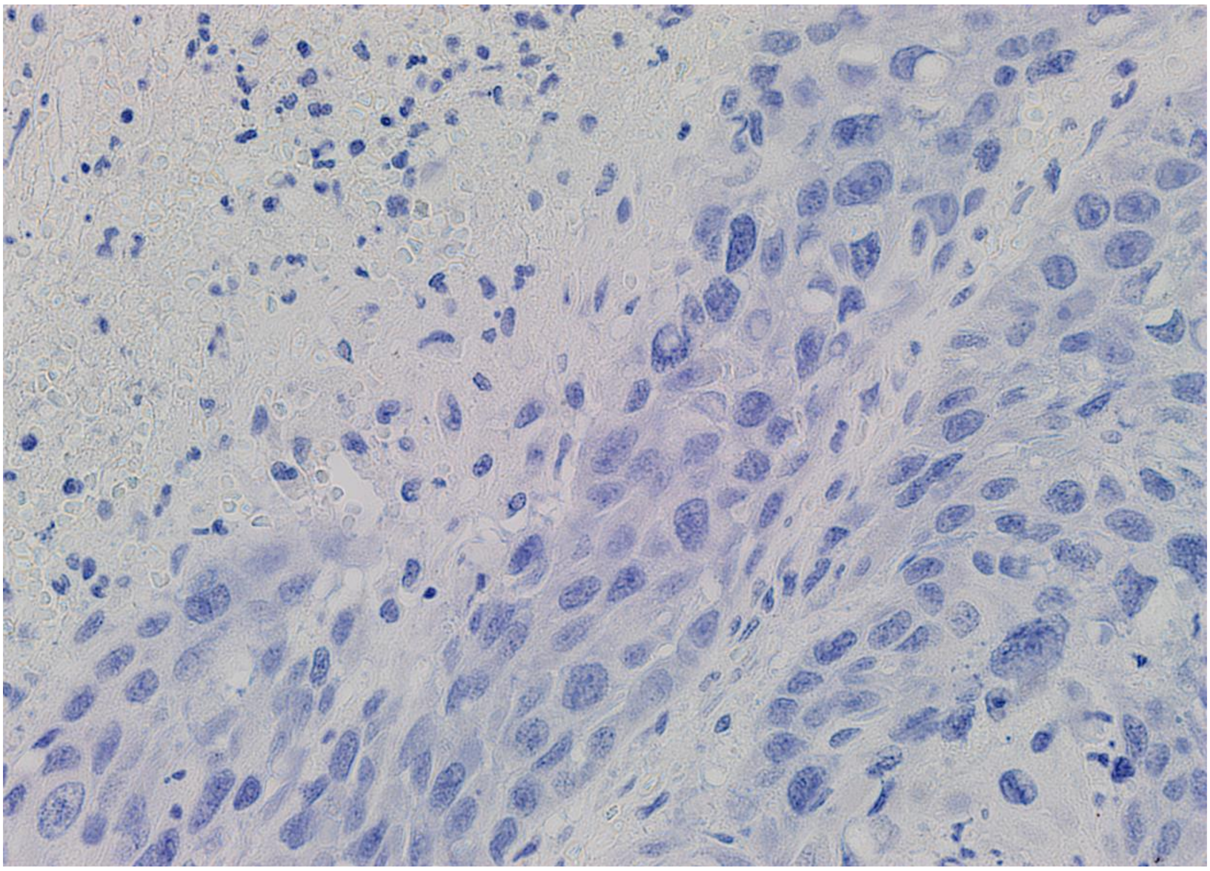

Figure S2. DAB IHC-stained section for MDLSCC with omission of the primary antibody as an appropriate negative control, demonstrating the absence of any non-specific staining (brown). Cellular nuclei were counterstained with hematoxylin (blue). Original magnification: 200x.

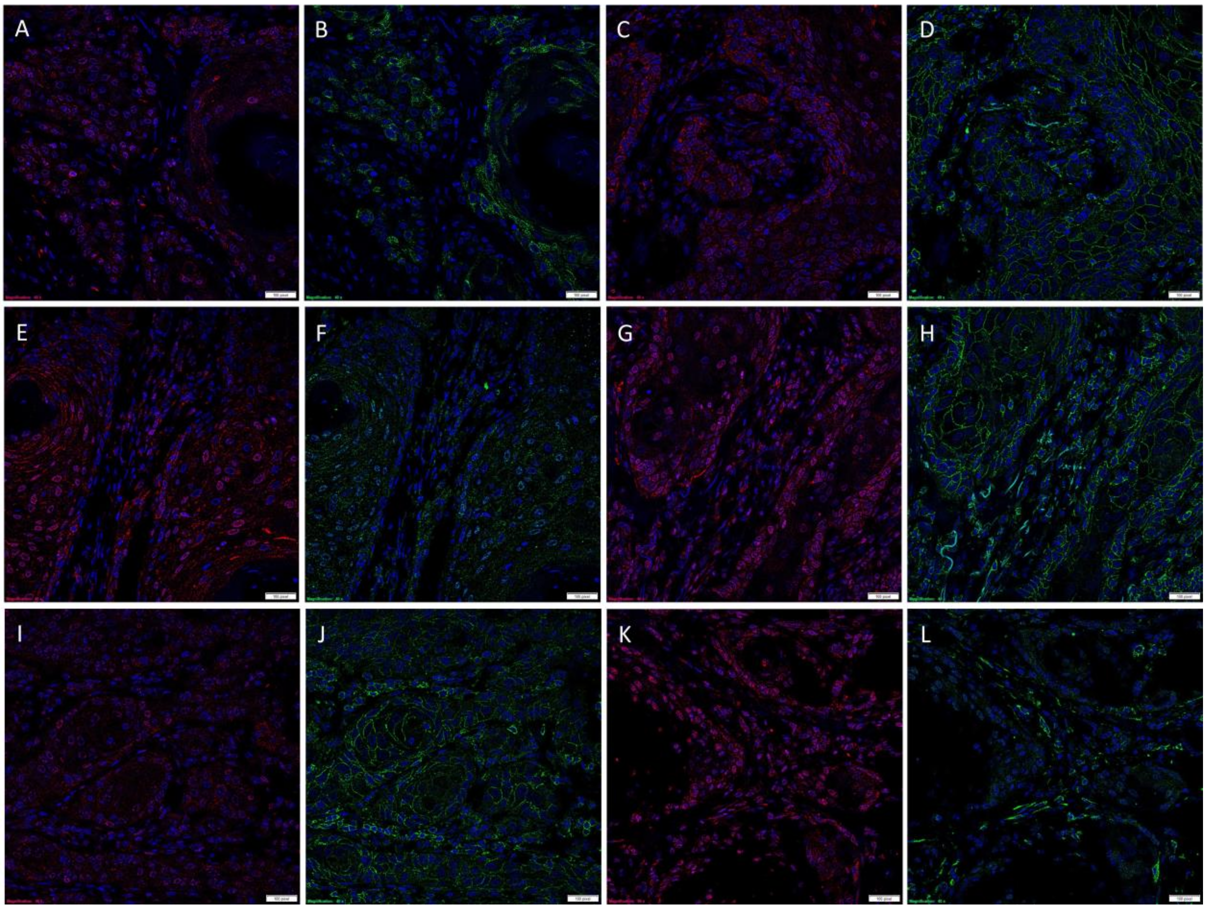

Figure S3. Split IF IHC-stained sections of moderately differentiated lip SCC shown in Figure 2 demonstrating nuclear expression of SOX2 (**A, C&E**, red), EMA (**B**, green), CD44 (**D**, green), SALL4 (**F**, green), NANOG (**G**, red), CD44 (**H**, green), pSTAT3 (**I**, red), SOX2 (**K**, red), and OCT4 (**L**, green). Cell nuclei were counterstained with 4',6-diamidino-2-phenylindole (**A-L**, blue). Scale bars: 20μm.
